# Gustatory Sensitivity and Food Acceptance in Two Phylogenetically Closely Related Papilionid Species: *Papilio hospiton* and *Papilio machaon*

**DOI:** 10.1371/journal.pone.0100675

**Published:** 2014-06-23

**Authors:** Giorgia Sollai, Iole Tomassini Barbarossa, Carla Masala, Paolo Solari, Roberto Crnjar

**Affiliations:** Department of Biomedical Sciences, Section of Physiology, University of Cagliari, Cagliari, Italy; AgroParisTech, France

## Abstract

In herbivorous insects, food selection depends on sensitivity to specific chemical stimuli from host-plants as well as to secondary metabolites (bitter) and to sugars (phagostimulatory). Bitter compounds are noxious, unpalatable or both and evoke an aversive feeding response. Instead, sugars and sugar alcohols play a critical role in determining and enhancing the palatability of foods. We assumed that peripheral taste sensitivity may be related to the width of the host selection. Our model consists of two closely phylogenetically related Papilionid species exhibiting a difference in host plant choice: *Papilio hospiton* and *Papilio machaon*. The spike activity of the lateral and medial maxillary styloconic taste sensilla was recorded following stimulation with several carbohydrates, nicotine and NaCl, with the aim of characterizing their gustatory receptor neurons and of comparing their response patterns in the light of their different acceptability in feeding behaviour. The results show that: a) each sensillum houses phagostimulant and phagodeterrent cells; b) the spike activity of the gustatory neurons in response to different taste stimuli is higher in *P. hospiton* than in *P. machaon*; c) sugar solutions inhibit the spike activity of the deterrent and salt cells, and the suppression is higher in *P. machaon* than in *P. hospiton.* In conclusion, we propose that the different balance between the phagostimulant and phagodeterrent inputs from GRNs of maxillary sensilla may contribute in determining the difference in food choice and host range.

## Introduction

All animals have taste chemoreceptor cells that respond to different food chemicals and the integrated activity of these cells plays a role in the balance between appetitive or aversive behaviour to foods. In fact, peripheral taste sensitivity plays a primary role in the choice of food both in invertebrates and vertebrates, including humans [1]–[11]. Herbivorous insects, and in particular the larvae of Lepidoptera, represent a suitable model to study the relationship between sensory input and behavioural output in the choice of food, as they exhibit clear food preferences and possess a limited number of gustatory neurons, housed within sensilla in the maxillae and epipharynx [12], [13]. Most of the electrophysiological studies have been focused on the two styloconic sensilla of each maxillary galea, since they are readily accessible. Both of these sensilla contain four gustatory receptor neurons (GRNs), the axons of which project directly to the central nervous system [4]. Each GRN responds to a limited range of compounds and the number of species for which the specific response profiles of these neurons are known, is still small [13], [14]. The species mostly investigated are *Pieris* sp., *Helicoverpa* sp., *Spodoptera* sp., *Grammia geneura*, *Bombyx mori* and *Manduca sexta*. In general, each lateral and medial sensillum has at least one sugar sensitive and one deterrent cell; the specific stimuli for the other cells are species dependent and include inositol, aminoacids, water and/or salts, as reviewed by Schoonhoven and van Loon [13]. Although cell identification is crucial to understand how sensory inputs interact in the process of food selection [13], information about the response specificities of the gustatory neurons in the peripheral taste system of Papilionid larvae is still lacking or dated [15], [16]. In herbivorous insects, and in particular in lepidopterous larvae, the differences in food selection between species are based on different central processing of sensory inputs, by means of post-ingestive mechanisms, or on differences in their gustatory systems, each adapted to a particular diet [6], [13]. These two models can be combined in a third model: the sense of taste and the central processing mechanism interplay closely to direct the insect towards the right host-plant [13]. Schoonhoven and van Loon [13] suggested that a comparative study on taste sensitivity in phylogenetically related monophagous and polyphagous species could answer the question whether feeding behaviour and food choices are related to peripheral sensitivity or to central processes.

On the basis of these considerations, in this work we performed experiments on the peripheral gustatory system of two phylogenetically related species of Lepidoptera (Papilionidae) [17]: *Papilio hospiton* Géné, endemic of the islands of Sardinia and Corsica and the Sardinian population of the Holarctic species *Papilio machaon* L. The two species are oligophagous, using various plants in the Apiaceae and Rutaceae families as hosts, and larvae do not feed on plants outside of these two families. In Sardinia, larvae of *P. machaon* are found on several Apiaceae, most frequently on *Foeniculum vulgare*, and on a few Rutaceae. Instead, for *P. hospiton*, *Ferula communis* is an almost exclusive host plant: only if *F. communis* is unavailable two other plants are used, one narrow endemic (*Ferula arrigonii*) and the other rare (*Ruta lamarmorae*) (unpublished data). This suggested the idea that *P. hospiton* is more specialized in host choice than *P. machaon*. Host specificity of lepidopteran insects is determined not only by female oviposition preferences, but also by larval food acceptance. In some cases, larvae may have no choice and need to adapt to the plant where they hatched. In this respect we considered that the larval peripheral taste sensitivity plays an important role in feeding acceptance governed by the balance between phagostimulant and phagodeterrent inputs.

We hypothesized that the different acceptability for food plants between the two species could reflect differences in the sensitivity profiles of their gustatory receptor neurons. To this end, we first stimulated both styloconic sensilla with sugars, one sugar alcohol, salts and nicotine, one plant alkaloid that humans taste bitter, to provide a functional characterization of each GRN in both species. Secondly, we evaluated qualitative and quantitative differences in the response profiles of GRNs between the two species. Third, we studied the presence of inhibitory effects by sugar solutions on the gustatory neurons activated by aversive concentration of salt. There is evidence from caterpillars of other species that sugars suppress the responses of the bitter and salt sensitive cells, thus making foods more appetitive [6], [13], [18]. Finally, we examined whether the peripheral interactions between neighbouring taste cells could be reflected in a different feeding behaviour between the two species. Upon contact with a mixture of compounds, the responses of neurons within the sensillum do not necessarily reflect their responses to the individual compounds alone: interactions may take place between the neurons after bioelectrical events have been initiated. Peripheral interactions are biologically meaningful because it is known that they are critically important in the normally feeding insect. In fact, interactions between neurons at the periphery will have the effect of altering the phagostimolatory or deterrent inputs, thus changing the balance and, accordingly, governing the behavioural responses of the insect [3].

## Materials and Methods

### Insects and rearing

*Papilio hospiton* Géné larvae were obtained from eggs laid in the butterfly oviposition annex (a 3×3×3 m cage) of the Physiology laboratories (University of Cagliari) by lab stock adult females on potted host-plants (*Ferula communis* L.); *Papilio machaon* L. was instead collected as eggs or 1^st^-3^rd^ instar larvae on wild fennel (*Foeniculum vulgare* Mill.) in the spring of 2012 in Cagliari (Sardinia, Italy). Caterpillars were reared and maintained at the insectary annex of the Physiology laboratories (University of Cagliari) in 1500-ml plastic cups (4–5 per cup) kept in an environmental growth chamber (24–25°C, 70% R.H., 16 h light/8 h dark photoperiodic regime) and checked daily until fit for the experiments.

Caterpillars were raised from eggs on their specific host-plants: *F. communis* for *P. hospiton* and *F. vulgare* for *P. machaon*. Fresh foliage was provided everyday and was available ad libitum. In order to have fresh host-plant available daily, several ferula plants were grown in the yard nearby the butterfly cage, while wild fennel was collected in the fields around the University campus.

### Electrophysiological experiments

Electrophysiological recordings were performed on 5th instar larvae two days after moulting [19] from the medial and lateral maxillary styloconic sensilla. Spike activity from chemosensory cells of either sensillum type was recorded by means of the “tip-recording” technique [20]. The reference electrode, a thin Ag/AgCl, was inserted into the head and gently pushed into the maxillary-labial complex to fix the maxillae in a prognathous position. The recording electrode, a glass micropipette (tip diameter 20 µm), filled with the stimulating solution, was placed over the sensillum tip. All signals were recorded with a high input impedance (10^15^ Ω) electrometer (WPI, Duo 773), band-pass filtered (0.1–3 KHz), digitized by means of an Axon Digidata 1440A A/D acquisition system (sampling rate 10 KHz) and stored on PC for later analysis.

### Electrophysiological stimuli

For all experiments, taste solutions were prepared immediately before testing and were presented at room temperature. The chemical stimuli were purchased from Sigma-Aldrich, (Italy). Medial and lateral sensilla were tested with KCl (50 mM), NaCl (1, 10, 100, 500 mM), nicotine (0.1, 1, 10 mM), *myo*-inositol (0.1, 1, 10 mM), glucose, fructose and sucrose (1, 10, 100, 250 mM). Although a higher concentration of salt stimulates deterrent cells and induces aversive behaviour in the two species, 50 mM KCl was used to dissolve all compounds except for NaCl, with the aim of optimizing recording conditions and signal-noise ratio for better spike identification [4], [6], [10]–[12], [14], [21]–[36].

Stimuli were applied in a randomized sequence except for 50 mM KCl that was tested first (control solution). A 3-min interval was allowed between consecutive stimulations to minimize adaptation phenomena. At the end of each sequence, 50 mM KCl was tested again to assess any shift in responsiveness; whenever relevant spike frequency variations were found (wider than 50%), the experiment was discarded: this occurred in less than 10% of the experiments.

In order to avoid any drift in solution concentration due to evaporation, a clean, dry piece of filter paper was used to draw fluid from the tip of recording/stimulating electrode just before each stimulation. After each stimulation, the mouthparts of the insect were rinsed with distilled water and blotted dry. Finally, we recorded only from sensilla on one side of each caterpillar (*N* = 26 for *P. hospiton* and *N* = 24 for *P. machaon*) and no caterpillar was used in more than one experiment.

### Data analysis

**Figure 1:**
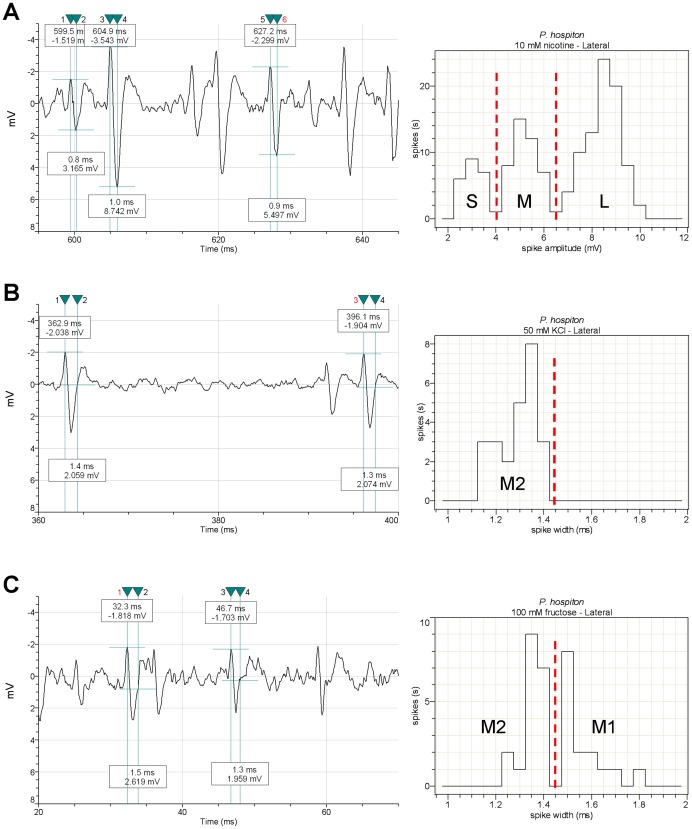
Identification of GRNs in the lateral sensillum by sorting of spike types. A) Spike identification by amplitude (Clampfit 10.0 software). Three different spikes are shown: “L”, “S” and “M” in the trace sample in response to 10 mM nicotine (left). Spike amplitude classes are given in the histogram (right); B) and C) Sorting of spike “M” by width (duration) measured as the spike end-to-peak time (Clampfit 10.0 software) in response to 50 mM KCl alone and 100 mM fructose, respectively. Sample traces with cursors indicating the time frame measured at left. Spike width classes are given in the histograms at right. The sorting analysis yields two different “M” spikes, named “M1” and “M2”. Vertical red dashed lines are the ideal boundaries of the spike types. All traces shown are from *P. hospiton*.

**Figure 2:**
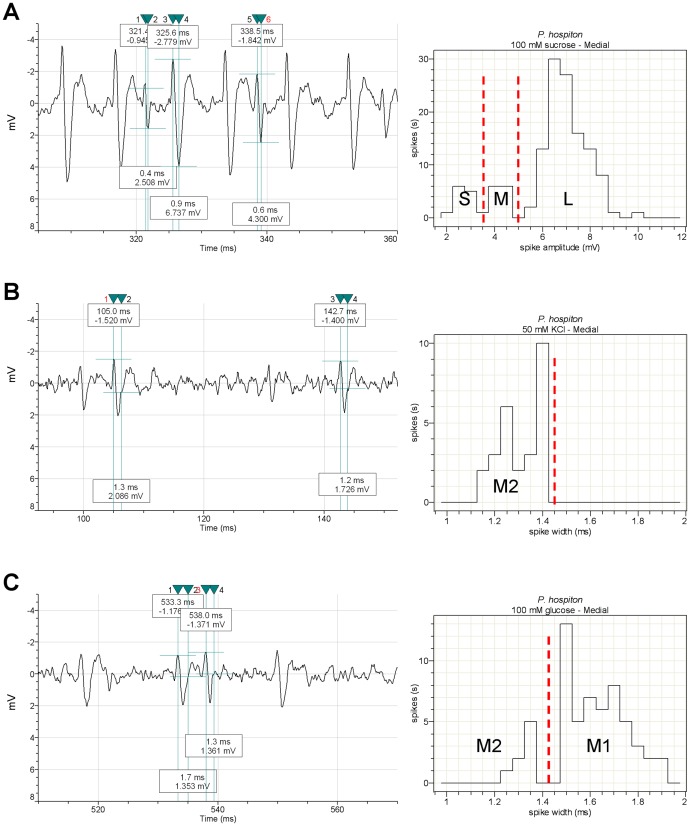
Identification of GRNs in the medial sensillum by sorting of spike types. A) Spike identification by amplitude (Clampfit 10.0 software). Three different spikes are shown: “L”, “S” and “M” in the trace sample in response to 100 mM sucrose (left). Spike amplitude classes are given in the histogram (right); B) and C) Sorting of spike “M” by width (duration) measured as the spike end-to-peak time (Clampfit 10.0 software) in response to 50 mM KCl alone and 100 mM glucose, respectively. Sample traces with cursors indicating the time frame measured at left. Spike width classes are given in the histograms at right. The sorting analysis yields two different “M” spikes, named “M1” and “M2”. Vertical red dashed lines are the ideal boundaries of the spike types. All traces shown are from *P. hospiton*.

Sensory recordings typically lasted 2–3 s, but spike sorting was performed in the interval 10–1010 ms after contact with the sensillum, the first 10 ms being skipped as containing the contact artifact. The 1st second of the discharges was chosen as representative of the phasic/phasic-tonic parts of the response [27], [30], [32], [37]. A preliminary sorting of action potentials was performed, on the basis of their amplitudes, by means of the VIEWDAT SAPID Tools [38]. For both species we identified three spike types that were labeled: small (S), large (L) and intermediate (M), in response to nicotine in the lateral sensillum and to fructose and/or sucrose in the medial sensillum. The results of this visual inspection were subsequently confirmed by measuring the peak-antipeak amplitude of action potentials, by means of the Clampfit 10.0 software as shown in Figures 1 and 2. We then characterized the responses to KCl alone, added as a conducting agent to all stimulus solutions, except for NaCl. Visual inspection of the recordings in response to KCl showed that both spike types previously identified as “M” and “S” were present. Spike “M” was found to respond to all sugars (+KCl) in the lateral sensillum and to glucose and inositol (+KCl) in the medial sensillum at a higher frequency than to KCl alone. This observation, together with the fact that each sensillum is known to contain four GRNs [13], led us to suppose that two different cells, firing spikes of the similar amplitude, were responding to these sugars. The parameter chosen to verify this hypothesis was the time interval between the end and the peak of the action potential, measured by the Clampfit 10.0 software. The duration of the “M” spike measured in the responses to KCl was in the range 1.2 ÷ 1.4 ms, while in response to sugars it fell into two separate ranges: 1.2 ÷ 1.4 ms and 1.5 ÷ 1.9 ms, that were assigned to two different classes by the Clampfit 10.0 software (Figs. 1B, C; 2B, C; see also figures S1–S9 in file S1). We then labelled these two spike types “M2” and “M1”, respectively. Parameters and software adopted for spike sorting in this study are based on earlier studies [39]–[44].

### Behavioural experiments

The feeding assay protocol was adapted from Glendinning et al. [6], [45] and involves five steps. (a) The test caterpillar is placed in an arena consisting of an inverted Petri dish covered with a clear plastic cylinder (13 cm in diameter and 7 cm tall) where it lies deprived of food for 30 min in order to standardize its “hunger” state. (b) The caterpillar is transferred to the “test-arena”, identical to the previous one except for a piece of cork (1 cm in diameter, 4–5 mm high) taped to the center of the inverted Petri dish. Immediately before each test session, a glass-fiber disk (Whatman GF/A, 4.25 cm in diameter; Sigma-Aldrich, Italy) was pinned to the cork, and then moistened with 400 µl of control or test solution. (c) the caterpillar is positioned on the edge of the disk and the assay starts when the caterpillar taps the disk surface with its chemosensilla. (d) At the end of a 2 min feeding period, the caterpillar is removed from the “test-arena” and is transferred to a plastic cup for 30 min, where it has ad libitum access to its host-plant. (e) Finally, the caterpillar is returned to the “food-deprivation arena” for 30 min, to start a new testing cycle. Each larva was tested with all stimuli at both concentrations (see below).

To evaluate feeding behaviour we measured two parameters: (a) the latency to start feeding, as the time elapsed between initial tasting the surface of the glass-filter disk and initiating feeding and (b) the total amount of disk area eaten during the 2 min feeding assay. To evaluate the disk area eaten we calculated the differences between the dried weight of each disk moistened with 400 µl of a test stimulus before (control) and after a 2 min feeding assay.

### Behavioural stimuli

The following taste stimuli were tested: fructose, glucose, sucrose (10, 100 mM), *myo*-inositol (1, 10 mM), KCl (50 mM) and bidistilled water. All stimuli were dissolved in 50 mM KCl, like the test solutions used in the electrophysiological recordings, or in bidistilled water, as a control in respect to KCl.

### Statistical analysis

Repeated-measures ANOVA was used: a) to analyze, in both species, the effect of increasing concentrations of taste stimuli (nicotine, inositol, sucrose, glucose, fructose and NaCl) on the spike frequency in the first second of discharges of GRNs (“L”, “M1”, “M2” and “S”) of the lateral and medial sensilla, separately for each taste stimulus (fixed factor: species (2 levels); repeated measures factor: concentration (3 levels for nicotine and inositol, 4 levels for sugars and NaCl); b) to compare differences, between the two species, of the spike frequency in the first second of discharges of GRNs (“L”, “M1”, “M2” and “S”) of the lateral and medial sensilla in response to high concentrations of stimuli for which a dose-response relationship was found in each GRN (three fixed factors: species (2 levels), sensillum type (2 levels) and GRN type (4 levels); repeated measures factor: stimuli (7 levels); c) to analyze the inhibitory effect of increasing concentration of sugars on the spike frequency of the “M2” and “S” GRNs evoked by 50 mM KCl, separately for each GRN (fixed factor: species (2 levels); repeated measures factor: concentration (4 levels for inositol, 5 levels for sugars); d) to analyze the effect of the interaction between sugars and KCl on feeding latency (fixed factor: species (2 levels); repeated measures factor: feeding substrates (16 levels) and on amount of food eaten (fixed factors: species (2 levels), feeding substrates (16 levels); repeated measures factor: before and after feeding (2 levels). Data were checked for the assumptions of homogeneity of variance, normality and sphericity (when applicable). When the sphericity assumption was violated, a Greenhouse-Geisser correction or Huynh-Feldt correction was applied in order to modify the degrees of freedom.

Post-hoc comparisons were conducted with the Tukey test, unless the assumption of homogeneity of variance was violated, in which case the Duncan's test was used. Statistical analyses were made using STATISTICA for WINDOWS (version 7.0; StatSoft Inc, Tulsa, OK, USA). *P* values<0.05 were considered significant.

### Permits

Required permits were obtained for *Papilio hospiton*. Specimens were collected in Sardinia in the spring of 2012, in compliance with the permit issued on 28 May 2012 (Ref. # 0010888) to Roberto Crnjar and his collaborators, by “Ministero dell'Ambiente e della Protezione del Territorio e del Mare” (Italian Board of Environment and Protection of Land and Sea), in derogation from the provisions set out in the regulation DPR 357/97 concerning the application of the “Council Directive 92/43/EEC of 21 May 1992 on conservation of natural habitats and of wild fauna and flora”. No specific permits were required for *Papilio machaon* and host-plants (*Ferula communis* and *Foeniculum vulgare*), as they are not endangered or protected species. Both plants and *P. machaon* were collected on public land.

## Results

### Functional characterization of gustatory receptor neurons (GRNs) of the lateral and medial styloconic sensilla

**Figure 3:**
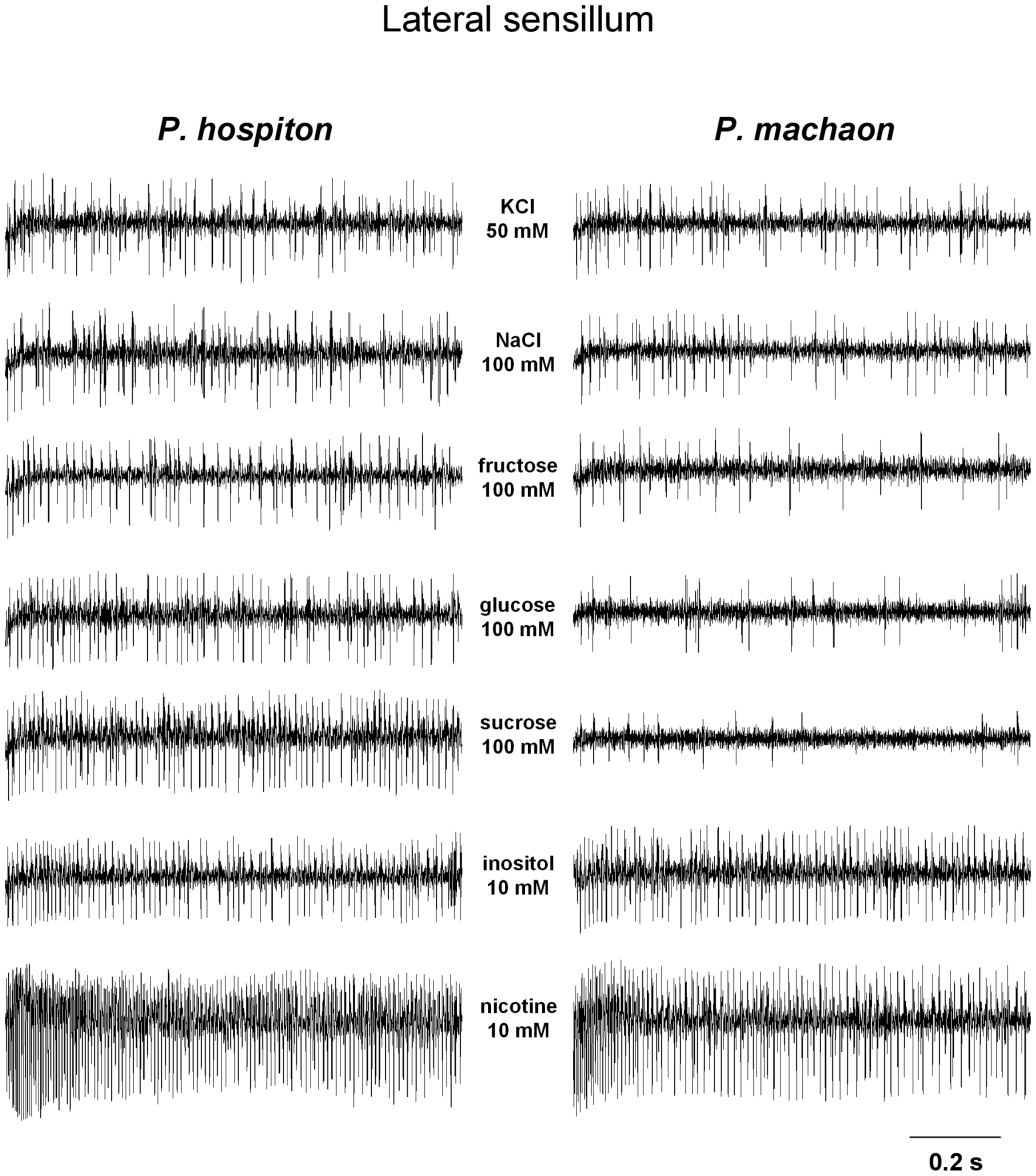
Sample of spike discharges of a lateral sensillum in both species. Sample traces showing spike firing frequency of a lateral styloconic sensillum following stimulation with KCl (control), NaCl, fructose, glucose, sucrose, inositol and nicotine.

**Figure 4:**
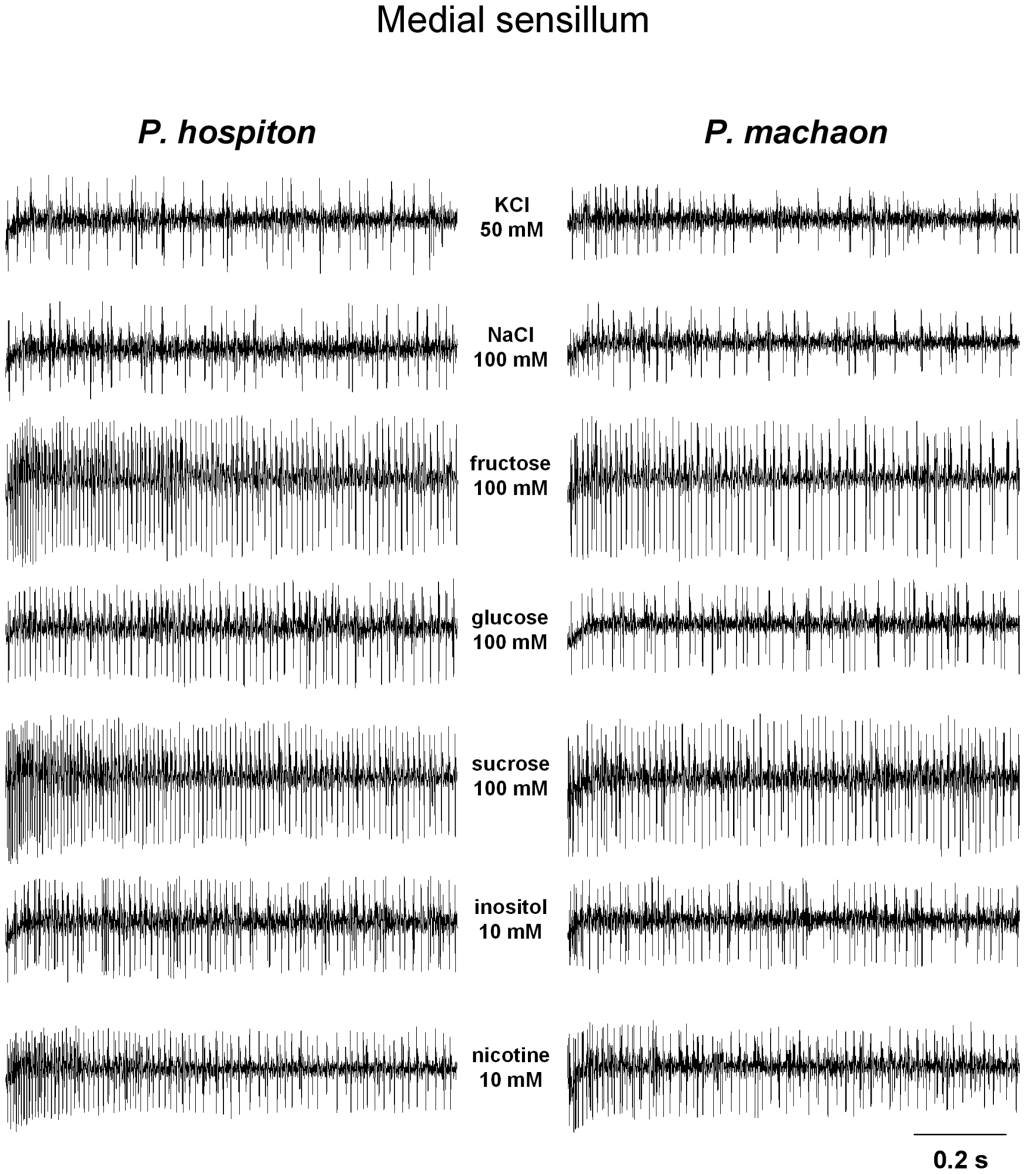
Sample of spike discharges of a medial sensillum in both species. Sample traces showing spike firing frequency of a medial styloconic sensillum following stimulation with KCl (control), NaCl, fructose, glucose, sucrose, inositol and nicotine.

Samples of spike discharges of the activity of different GRNs, recorded from the lateral and medial styloconic sensilla of both species, in response to chemicals tested are shown in Figs. 3, 4.

To test for a dose-response relationship, we analyzed the spike activity evoked in the first second of the discharge for each GRN (“L”, “M1”, “M2” and “S”) to increasing concentrations of nicotine, several carbohydrates and NaCl, in both sensilla and species, by using a repeated-measures ANOVA.

**Figure 5:**
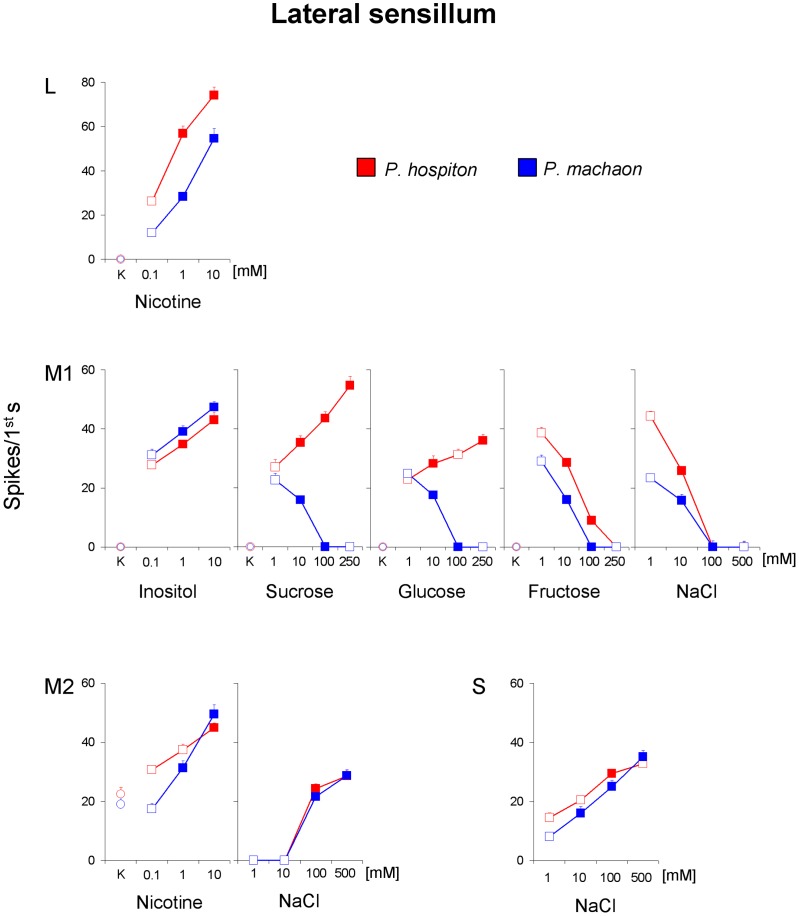
Functional characterization of GRNs of the lateral styloconic sensillum in *P. hospiton* and *P. machaon*. Dose-response relationship between spike activity of GRNs and different taste stimuli. All values are mean (± s.e.m.); *N* = 26 for *P. hospiton*, *N* = 24 for *P. machaon*. Filled symbols indicate significant differences between a concentration and that next lower (*P*<0.05). Circle symbols indicate the GRN responses to 50 mM KCl (K).

For the lateral styloconic sensillum (Fig. 5), repeated-measures ANOVA showed for both species a significant effect of concentration on the spike frequency of the “L” GRN in response to nicotine (*F*_[2,96]_ = 117.44; *p*<0.00001), and post-hoc comparisons showed that the spike frequency in response to each concentration was higher than in response to the next lower concentration (*p*<0.0001; Duncan's test). These results, together with the analysis of the neural traces (Fig. 3), indicate that, in both species, “L” neuron is activated only by nicotine. Repeated-measures ANOVA also showed for both species a significant effect of concentration on the spike frequency of the “M1” GRN in response to inositol (*F*_[2,96]_ = 34.947; *p*<0.00001), and post-hoc comparisons that the neural activity in response to each concentration was higher than in response to the next lower concentration (*p*<0.05; Tukey test). Repeated-measures ANOVA revealed a significant two-way interaction of Concentration × Species on the spike frequency of the “M1” GRN in response to sucrose and glucose (*F*_[3,144]_>59.9; *p*<0.00001). Pairwise comparison showed, in *P. hospiton*, increases of spike activity in response to sucrose and glucose for each concentration step (*p*<0.001 and *p*<0.05, respectively; Duncan's test). On the contrary, post-hoc comparisons showed, in *P. hospiton*, decreases of spike activity in response to fructose and NaCl, and in *P. machaon* to sucrose, glucose, fructose and NaCl (for each concentration step; *p*<0.005; Tukey test). These findings, together with the analysis of spike traces (Fig. 3), indicate that a same single taste neuron (“M1”) is activated by different carbohydrates in *P. hospiton* and only by inositol in *P. machaon*. Moreover, repeated-measures ANOVA revealed a significant two-way interaction of Concentration × Species on the spike frequency of the “M2” GRN in response to nicotine (*F*_[2,96]_ = 7.5633; *p*<0.001), and post-hoc comparison showed significant increase in response to nicotine 10 mM in *P. hospiton* (*p*<0.01; Tukey test), while already to 1 mM in *P. machaon* (*p*<0.01; Tukey test). Repeated-measures ANOVA also showed for both species a significant effect of concentration on the spike frequency of the “M2” GRN in response to NaCl (*F*_[3,144]_ = 7.5633; *p*<0.001) and post-hoc comparisons revealed a significant increase of the response to 100 mM NaCl (*p*<0.001; Tukey test). These results, together with the analysis of the neural traces (Fig. 3), indicate that, in both species, “M2” neuron is activated by nicotine and salts at high concentrations. Finally, repeated-measures ANOVA showed a significant effect of concentration on the spike activity of the “S” GRN in response to NaCl (*F*_[3,144]_ = 68.049; *p*<0.0001) and pairwise comparisons a significant increase of spike frequency for each concentration step in *P. machaon* (*p*<0.01; Tukey test), while only at 100 mM in *P. hospiton* (*p*<0.005; Tukey test). No other concentration effects were found. These results and the analysis of spike discharges (Fig. 3), suggest that, in both species, “S” neuron is activated by inorganic salts.

**Figure 6:**
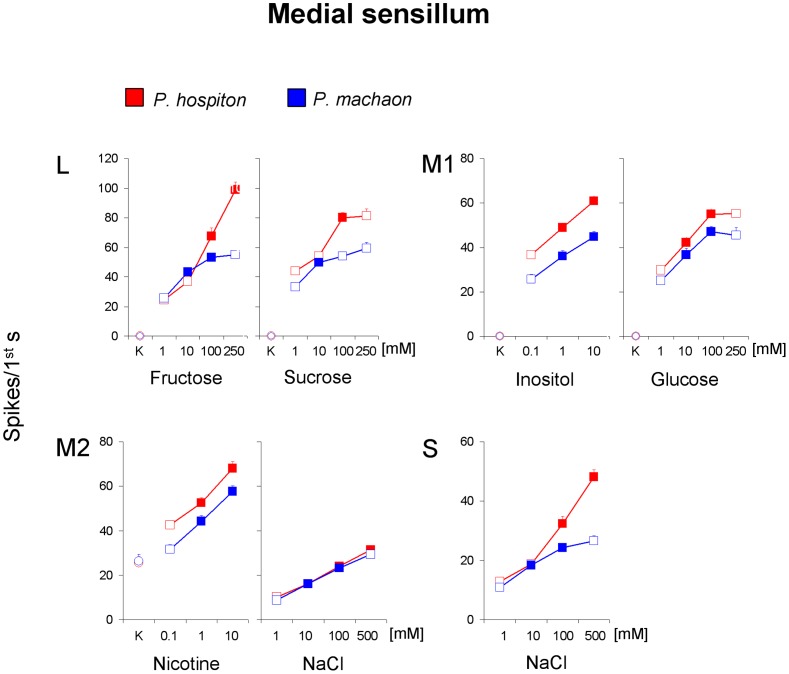
Functional characterization of GRNs of the medial styloconic sensillum in *P. hospiton* and *P. machaon*. Dose-response relationship between spike activity of GRNs and different taste stimuli. All values are mean (± s.e.m.); *N* = 26 for *P. hospiton*, *N* = 24 for *P. machaon*. Filled symbols indicate significant differences between a concentration and that next lower (*P*<0.05). Circle symbol indicate the GRN responses to 50 mM KCl (K).

For the medial styloconic sensillum (Fig. 6), repeated-measures ANOVA revealed a significant two-way interaction of Concentration × Species on the spike frequency of the “L” GRN in response to fructose and sucrose (*F*_[2.76,131]_>4.3706; *p*<0.00001) and post-hoc comparisons show that both sugars evoked a significant increase of spike frequency to the second concentration step in *P. machaon* (*p*<0.05, Tukey test), and to the third in *P. hospiton* (*p*<0.0001, Tukey test). Repeated-measures ANOVA revealed, in both species, a significant effect of concentration on the spike frequency of the “M1” GRN in response to inositol (*F*_[2,96]_ = 35.524; *p*<0.00001) and glucose (*F*_[3,144]_ = 41.415; *p*<0.00001), and post-hoc comparisons revealed that the response evoked by each concentration of inositol and glucose was higher than that by next lower (*p*<0.01, Tukey test), except for glucose 250 mM that was ineffective to determine a further increase of frequency (*p*>0.05). Besides, repeated-measures ANOVA revealed, in both species, a significant effect of concentration on the spike frequency of the “M2” GRN in response to nicotine (*F*_[2,96]_ = 53.889; *p*<0.00001) and NaCl (*F*_[3,144]_ = 60.807; *p*<0.00001), and post-hoc comparisons indicated that the response evoked by each concentration was higher than that by the next lower (*p*<0.05, Tukey test), except for 500 mM NaCl in *P. machaon* that was ineffective to determine a further increase of frequency (*p*>0.05). Finally, repeated-measures ANOVA revealed a significant two-way interaction of Concentration × Species on the spike frequency of the “S” GRN in response to NaCl (*F*_[2.88,138]_ = 19.357; *p*<0.00001) and post-hoc comparisons showed a significant increase of spike frequency to each concentration step (*p*<0.01; Tukey test), except for 500 mM in *P. machaon* that was ineffective to determine a further increase (*p*>0.05). No other concentration effects were found.

These results, together with the analysis of the neural traces (Fig. 4), indicate that, for the medial sensillum: “L” neuron is activated by fructose and sucrose; “M1” is activated by glucose and inositol; “M2” neuron is activated by nicotine and salts at high concentrations; “S” neuron is activated by inorganic salts.

### Differences between the two species of the electrophysiological responses for the lateral and medial sensilla GRNs to specific taste stimuli

**Figure 7:**
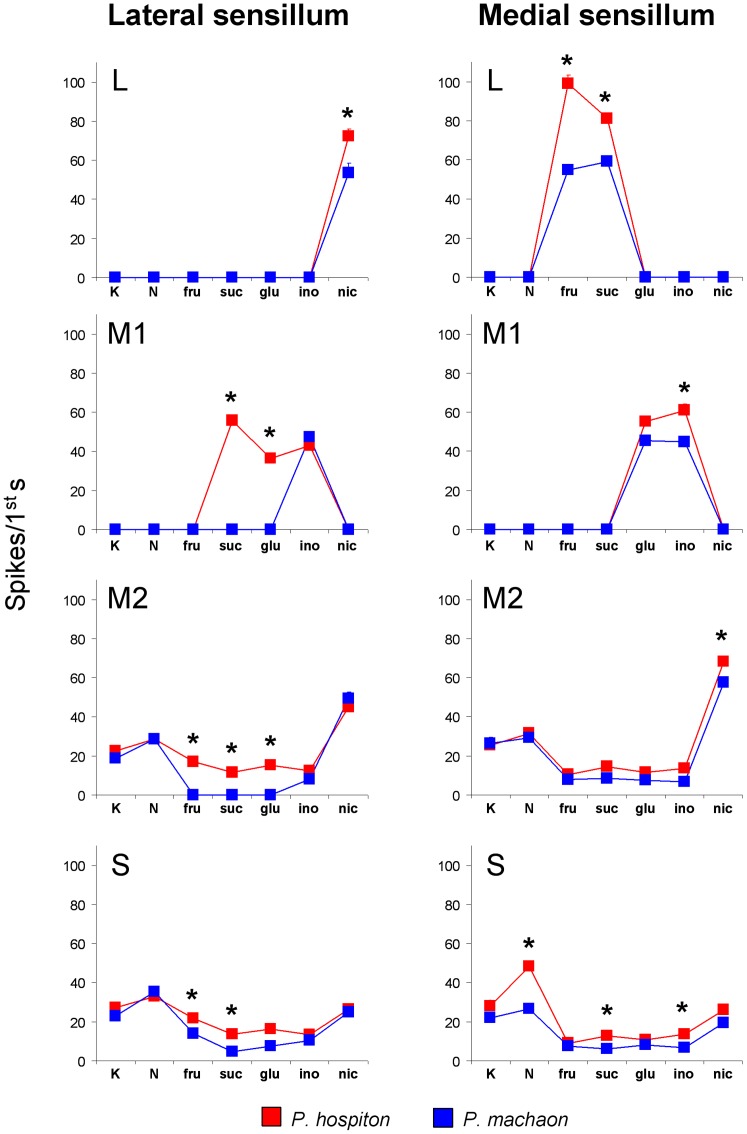
Differences between the two species of the electrophysiological response to specific taste stimuli of lateral sensillum GRNs. Mean values ± s.e.m. of spike frequencies of the lateral and medial “L”, “M1”, “M2” and “S” GRNs. K =  KCl (control); N: NaCl; fru: fructose; suc: sucrose; glu: glucose; ino: inositol; nic: nicotine. *N* = 26 for *P. hospiton*, *N* = 24 for *P. machaon*. (*) significant differences between the two species (*P*<0.05).

Repeated-measures ANOVA was used to test quantitative differences, between the two species, of the spike frequency in the first second of discharges by GRNs (“L”, “M1”, “M2” and “S”) of the lateral and medial sensilla in response to high concentrations of stimuli for which a dose-response relationship was found in each GRN (Fig. 7). For this analysis the responses to higher concentrations were chosen so as to exclude the “water” response component from the spike discharges. This could represent a confounding factor in the analysis of the discharges for the lateral “M1” GRN.

ANOVA showed a significant interaction of Stimulus × Species × Sensillum type × GRN on the spike frequency (*F*_[16,2049]_ = 26.895; *p*<0.00001). For the lateral sensillum, post-hoc comparisons showed that the spike frequency of “L” GRN in response to nicotine (*p* = 0.00005; Tukey test), of “M1” GRN in response to sucrose and glucose (*p*<0.000001; Duncan's test), of “M2” GRN in response to KCl added to fructose, sucrose and glucose (*p*<0.00005; Duncan's test), and of “S” GRN in response to KCl added to fructose and sucrose (*p*<0.005; Duncan's test), were higher in *P. hospiton* than in *P. machaon*. For the medial sensillum, post-hoc comparisons showed that the spike frequency of “L” GRN in response to fructose and sucrose (*p*<0.000001; Duncan's test), of “M1” GRN in response to inositol (*p* = 0.00005; Tukey test), of “M2” in response to nicotine (*p* = 0.000034; Duncan's test), and of “S” GRN in response to NaCl (*p*<0.000001; Tukey test) and to KCl added to the sucrose and inositol (*p*<0.05; Duncan's test), were higher in *P. hospiton* than in *P. machaon*. These results indicate that *P. hospiton* presents a higher peripheral taste sensitivity than *P. machaon*, to bitter, sugar and salt compounds.

No significant changes between the two species were found in the spike frequency evoked by the other stimuli for both sensilla (*p*>0.05; Tukey or Duncan's test).

The 50 mM KCl solution (control), that was included in the analysis as it is added to all stimulus solutions, but NaCl, to make them conductive, elicited a neural activity from “M2” and “S” GRNs in both sensilla and in both species (Fig. 7). In this case, no statistical differences were found between the two species.

### Inhibitory effect of increasing concentrations of sugars on the spike frequency of the “M2” and “S” GRNs in both species

**Figure 8:**
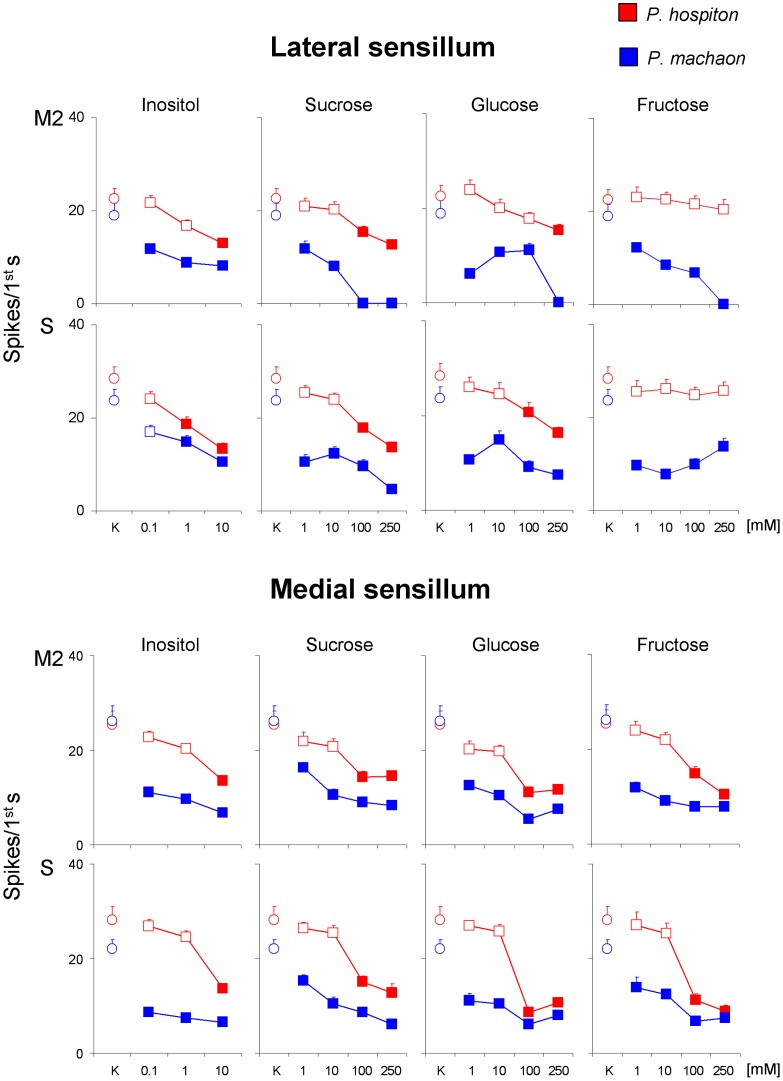
Inhibitory effect of increasing concentrations of sugars on the spike frequencies of the “M2” and “S” GRNs in both species. Mean values ± s.e.m. of spike frequencies of “M2” and “S” GRNs in the lateral and medial sensilla following stimulation with the control, 50 mM KCl (K; circles) and 50 mM KCl + increasing concentrations of each sugar (squares). *N* = 26 for *P. hospiton*, *N* = 24 for *P. machaon*. Filled squares indicate significant difference between the sugar concentration and the control (*P*<0.05).

To test for a lateral inhibition of sugar taste cell on the activity of deterrent and salt cells, we analyzed the effect of increasing concentrations of sugars, in both sensilla and species, on the spike activity of “M2” and “S” GRNs evoked by 50 mM KCl, by using repeated-measures ANOVA (Fig. 8). A significant interaction of Sugar concentration × Species was found on the activity of lateral “M2” GRN when sucrose, glucose and fructose were added (sucrose *F*_[3,144]_ = 4.2304; *p* = 0.0067, glucose *F*_[3,150]_ = 5.1788; *p* = 0.0017 fructose *F*_[3,140]_ = 4.7096; *p* = 0.0012), of medial “M2” GRN in the presence of fructose (*F*_[2.28,109]_ = 5.7348; *p* = 0.0029), and of medial “S” GRN when all sugars were added (inositol *F*_[2.34,112]_ = 9.6286; *p* = 0.000057, sucrose *F*_[3,146]_ = 2.9226; *p* = 0.035, glucose *F*_[2.7,129]_ = 9.7175; *p* = 0.000018, fructose *F*_[3.2,153]_ = 4.7243; *p* = 0.0029). Post-hoc comparisons showed that in *P. machaon* all sugars were effective on both GRNs of both sensilla at the lowest concentration used (*p*<0.01), except for inositol that was effective on “S” GRN of the lateral sensillum at 1 mM (*p* = 0.0042). On the other hand, in *P. hospiton*, inositol was effective on “M2” GRN of the lateral sensillum and both GRNs of the medial sensillum at the highest concentration (*p*<0.0001); sucrose was effective on both GRNs of both sensilla at 100 mM concentration (*p*≤0.005); glucose was effective on “S” GRN of the lateral sensillum and both GRNs in the medial sensillum at 100 mM (*p*≤0.0005; Tukey test), and at 250 mM in the case of “M2” GRN of the lateral sensillum (*p* = 0.0057); finally fructose was effective at 100 mM on both GRNs of the medial sensillum (*p*≤0.0005; Tukey test), while was ineffective on the lateral sensillum (*p*>0.05). These results indicate that the inhibitory effect of sugars was greater in *P. machaon* than in *P. hospiton* for both GRNs of both sensilla. The comparisons among species are valid because no statistical difference was found, between the two species, in the response to KCl of both “M2” and “S” cells.

### Effect of the interaction between sugars and 50 mM KCl on feeding behaviour

**Figure 9:**
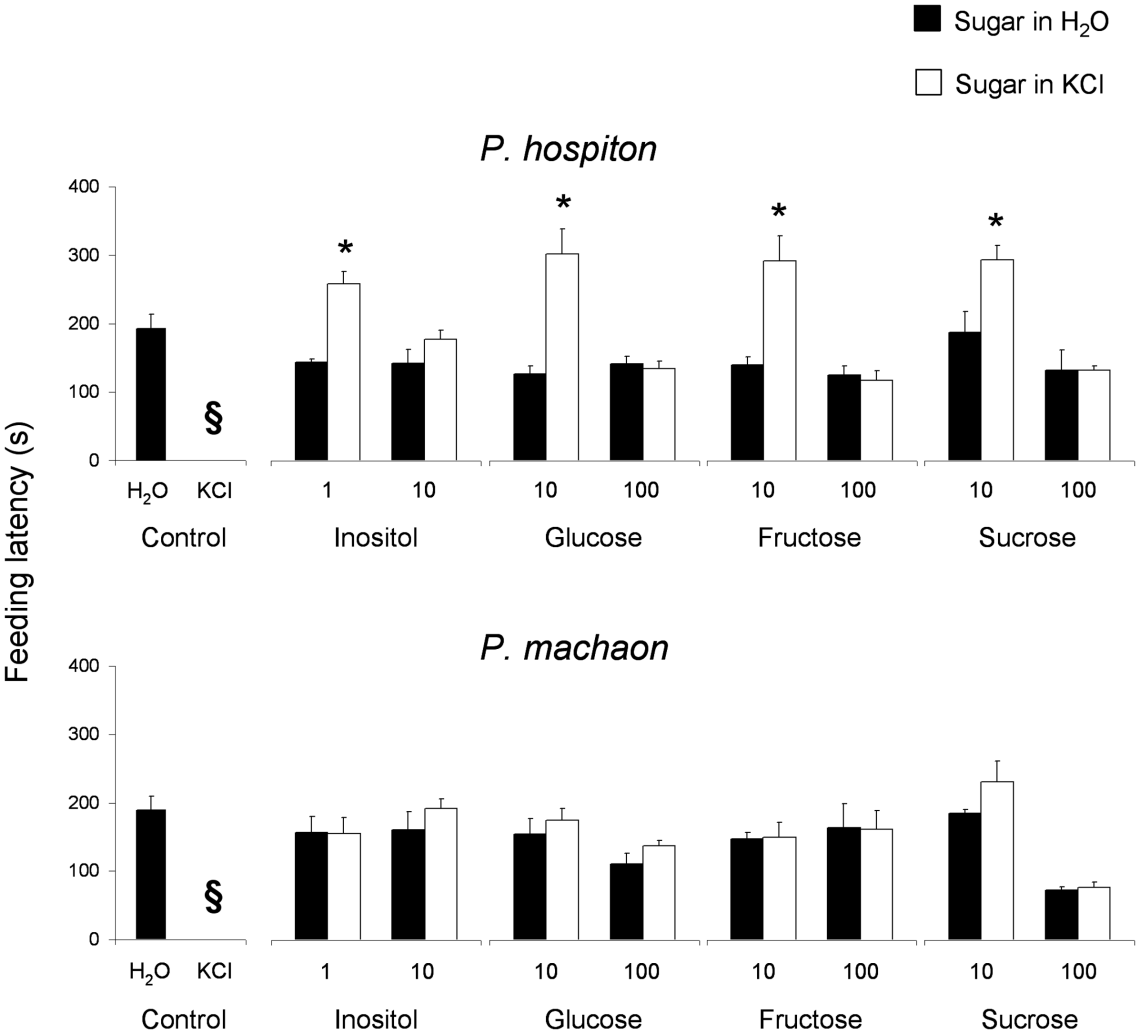
Effect of the interaction between sugars and KCl on feeding latency of both species. Mean values ± s.e.m. of time needed to caterpillars to start eating (feeding latency, s) glass-filter disks moistened with inositol (1 and 10 mM), glucose, fructose or sucrose (10 and 100 mM), dissolved in water or in KCl (50 mM), and with KCl (50 mM) or bidistilled water alone. *N* = 10 for both species. (*) significant differences (*P*<0.01) between sugar in water and sugar in KCl. (**§**) never started to feed.

Mean values ± s.e.m. of time needed to caterpillars of both species to start eating (feeding latency) glass-filter disks moistened with inositol (1 and 10 mM), glucose, fructose and sucrose (10 and 100 mM) dissolved in water or in KCl (50 mM), and with KCl (50 mM) or bidistilled water alone, are shown in Fig. 9. Repeated measures ANOVA revealed a significant interaction of Species × Feeding substrate on the feeding latency (*F*_[7,100]_ = 5.1723; *p*<0.00004). Post-hoc comparisons showed that, in *P. hospiton*, the feeding latency for the disks moistened with the low concentrations of sugars dissolved in KCl was higher than for disks imbibed with the corresponding sugar concentration in water (*p* = 0.00003 for inositol and glucose; *p* = 0.0001 for fructose; *p* = 0.01 for sucrose; Tukey test), while no differences were found for high concentrations. In the case of *P. machaon*, no differences were found (*p*>0.05).

**Figure 10:**
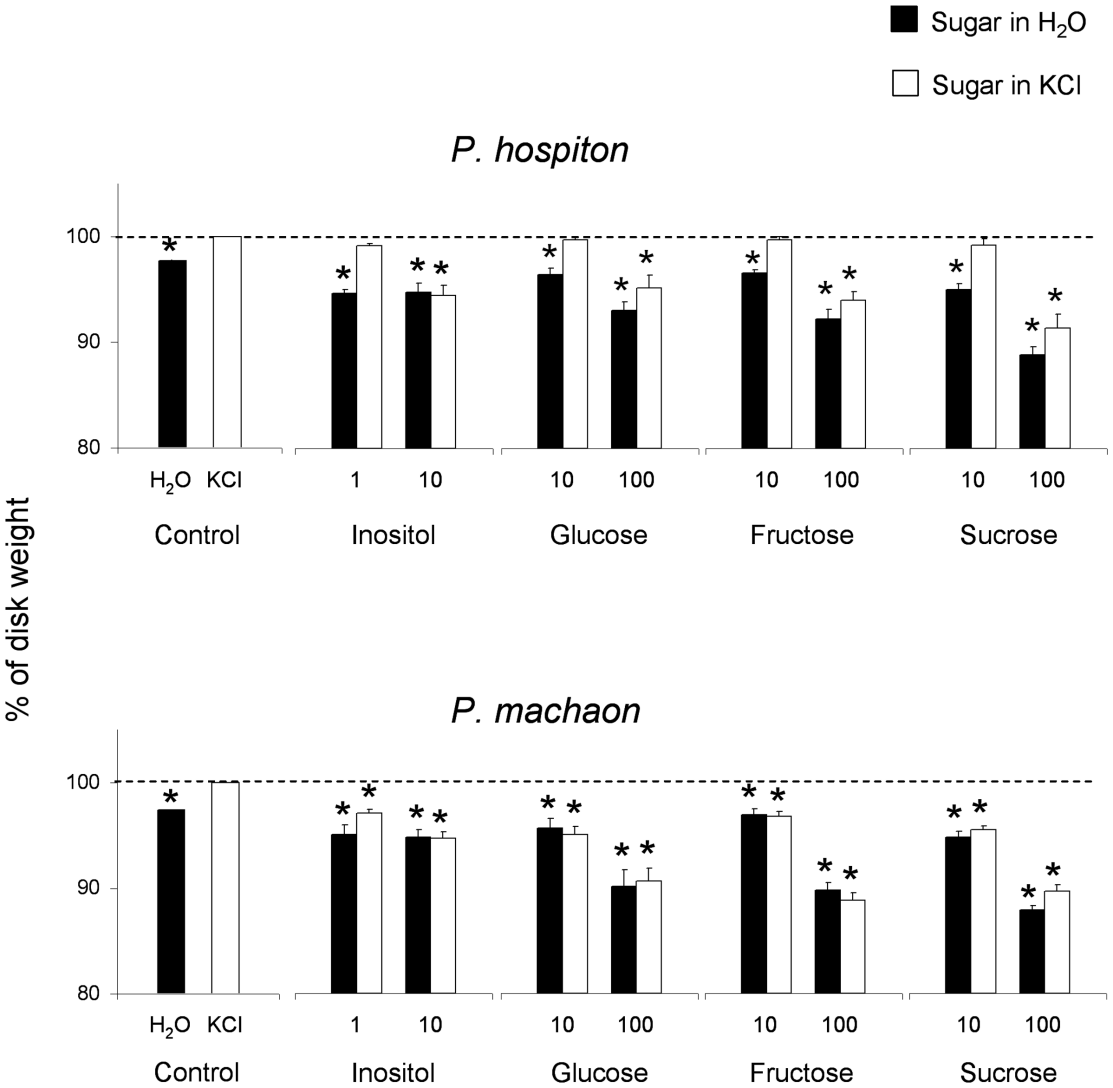
Effect of the interaction between sugars and KCl on amount of food eaten of both species. Percentage of remaining weight of glass-filter disks moistened with inositol (1 and 10 mM), glucose, fructose or sucrose (10 and 100 mM) dissolved in water or in KCl (50 mM), and with KCl (50 mM) or bidistilled water alone, after the 2-min feeding trial as compared to pre-trial values (control, 100%). *N* = 10 for both species. (*) significant differences between each sugar solution in water and the corresponding solution in KCl (*P*<0.05).

Fig. 10 shows mean values ± s.e.m. of percentage of remaining dried weight of glass-filter disks moistened with inositol (1 and 10 mM), glucose, fructose or sucrose (10 and 100 mM) dissolved in water or in KCl (50 mM), and with KCl (50 mM) or bidistilled water alone, for both species, after the 2-min feeding trial as compared to pre-trial values (control, 100% in the graphs). Post hoc comparisons showed, for each stimulus, that the disk weight differed statistically from the control when moistened with sugar solutions in water, for both *P. hospiton* and *P. machaon* (*p*<0.005 and *p* = 0.000146, respectively; Tukey test subsequent to repeated measures ANOVA). The sugar solutions dissolved in KCl were all appetible for *P. machaon* (*p*<0.005; Tukey test), while for *P. hospiton*, disk weight differed statistically from the control, only when moistened with high concentrations of sugars (*p*<0.04).

Finally, the results show that both species fed on glass-filter disks moistened with bidistilled water and in no case fed on those moistened with 50 mM KCl (Figs. 9, 10).

## Discussion

In all animals the main role of the peripheral gustatory system is to provide information about the nutritional properties of food and the presence of potentially harmful compounds. This function is performed by the gustatory receptor neurons (GRNs) that show specific responses toward different classes of chemical compounds. In this study we functionally characterize for the first time the GRNs of the lateral and medial styloconic sensilla in the larvae of two phylogenetically related Papilionid species having diverse feeding choices: *P. hospito*n and *P. machaon*. The dose-response relationships we found indicate that, in the lateral styloconic sensillum of both species, two GRNs out of four are specific cells (“L” and “M2”) to detect deterrent compounds, one is a salt cell (“S”) and one is specific to detect sugars and sugar alcohols (“M1”). The classification of “M2” as a deterrent cell that responds to nicotine and also to high concentrations of NaCl is in agreement with what reported both in vertebrates and invertebrates, where high salt concentrations stimulate the same cell as bitter compounds producing an aversive eating behaviour [7], [46]. The presence of one or more deterrent cells in larval styloconic sensilla has been reported in various species. In *Grammia geneura* Strecker (Lepidoptera: Arctiidae), both lateral and medial sensilla house two deterrent cells [14]. The lateral and medial sensilla of *Pieris brassicae* L. (Lepidoptera: Pieridae) contain one specialist deterrent cell and one generalist, respectively: the former causes food rejection by larvae, while the latter does not necessarily lead to rejection of the food source [47]. On the other hand, in *Manduca sexta* L. (Lepidoptera: Sphingidae) the discriminative taste processing between different bitter compounds that activate the same taste cell, is mediated by different temporal codes [29]. These reports suggest that the discrimination of various bitter compounds can be mediated either by means of different responsive cells or different temporal patterns of spike activity. Based on our electrophysiological results showing that two deterrent cells are located in the lateral sensillum, we assume that, in Papilionid larvae, two different neurons can contribute to the discriminative processing of different deterrent compounds. Since some bitter plant compounds (such as nicotine) are toxic while others are only unpalatable but harmless, we speculate that the “L” neuron is activated by toxic compounds (i.e. nicotine), while “M2” neuron either by toxic or other unpalatable compounds (such as inorganic salts at high concentrations), suggesting that inputs from both deterrent cells, but not from “M2” cell alone, could allow the insects to reject food.

Our results suggest a general phagostimulatory role for the “M1” cell; in fact, its responses are directly related to increasing concentrations of inositol (in both species), glucose and sucrose (in *P. hospiton*), and decreasing concentrations of fructose and NaCl (in both species) and glucose and sucrose (in *P. machaon*). There is evidence, in other vertebrates and invertebrates, that one same GRN may respond to different compounds, whether belonging or not to the same chemical class, although this is a still debated issue [48]. In mice, for instance, many taste receptor cells respond to both appetitive and aversive stimuli [2]. The medial sensillum of *G. geneura* has a single neuron with a general phagostimulatory function, as it responds to compounds belonging to three different chemical classes [23]. In the “i-type” sensilla of *Drosophila melanogaster* Meigen (Diptera: Drosophilidae) one same GRN responds to phagostimulatory compounds, such as sugars and low concentrations of NaCl [7]. Low concentrations of sugars and NaCl can be considered as a water stimulus, which is generally considered phagostimulant for insects [14], as responses of “M1” are inversely related to their concentrations, analogously to what reported in *Phormia regina* Meigen (Diptera: Calliphoridae), *D. melanogaster* and female adults of *Papilio xuthus* L. (Lepidoptera: Papilionidae) [49]–[51].

Information from the salt-S cell and the two antagonist GRNs, “M1” responding to low salt concentrations (water-sensitive phagostimulatory cell) and “M2” to high ones (salt-sensitive deterrent cell), suggests that the balance of the neural inputs from the two cells may help maintain appropriate salt consumption and regulate fluid and electrolyte homeostasis [14], [46], [52]. In fact, high salt concentrations activate “S” and phagodeterrent-M2 cells, allowing the insect to avoid ingestion of excess salt, while low salt concentrations activate “S” and phagostimulant-M1 cells, helping it to maintain an appropriate salt consumption. Thus, “S+M2” are involved in processing of aversive information, while “S+M1” in processing of appetitive information.

The medial styloconic sensillum of both *P. hospiton* and *P. machaon* houses two sugar cells (“L” and “M1”), one deterrent/salt cell (“M2”) and one salt cell (“S”). In fact, our dose-response results show that: “L” is activated by fructose and sucrose; “M1” is activated by glucose and inositol; “M2” is activated by nicotine and NaCl; “S” is activated by NaCl. The co-existence of two sugar-cells within the same sensillum, as well as a same cell capable of responding to both inositol and glucose, is an unusual finding; to the best of our knowledge, only in *M. sexta* the presence has been reported of a same carbohydrate cell activated by inositol and glucose, in both sensillum types, and of a second sugar cell, in the lateral sensillum [4], [30], while most herbivorous insects have one cell specific for inositol, its isomers and/or other sugar alcohols [10], [13], [14], [53], [54]. In *P. machaon* and *P. hospiton* inorganic salts stimulate two different neurons: one specific for salt and the other also responding to bitter compounds, as reported in other animals [14], [16], [55].

A primary aim of the present study was to determine whether differences in response patterns of the peripheral gustatory system of the two phylogenetically related Papilionid species, *P. hospiton* and *P. machaon*, can contribute to explain their different feeding choices. Our results highlight differences in peripheral neuronal responses between the two species. In fact, comparative analysis shows a different taste sensitivity to both bitter and sugar compounds between the two species. The stronger sensitivity to bitter compounds, that evoke an aversive behavioural feeding response [1], [45], could make *P. hospiton* more capable of avoiding potentially noxious food sources than *P. machaon*, thus playing a role in restricting the choice range of its host-plants. The effect the sugars have on the bitter and salt cells could contribute to better explain the feeding choice adopted by the two species. Further electrophysiological studies with other bitter compounds and behavioural experiments could be useful to elucidate this intricate matter. On the other hand, the difference in sensitivity profiles to sugars in phagostimulant cells of the two species suggests that they evaluate the appetitive plant sugars differently at the CNS level. Further studies are needed to determine whether a relationship exists between sensitivity profiles of phagostimulatory cells and host plant chemical profiles.

Previous reports suggest that the peripheral sensitivity to bitter and phagostimulatory compounds play a role in evoking an aversive or appetitive feeding behaviour, both in invertebrates and vertebrates, including humans [14], [16], [46]. In a comparative study between two related species of *Heliothis* (Lepidoptera: Noctuidae), one generalist and one specialist, Bernays et al. [1] examined behaviourally the sensitivity of caterpillars to several bitter compounds and they found that all compounds reduced feeding, but the generalist was less affected than the specialist. In *M. sexta* it has been suggested that the primary function of the peripheral taste response to sugars is to mask the taste of noxious compounds and to counteract the inhibitory input from the thoracic ganglion to the chewing movements of the mandibles [4], [12], [56]. Finally, sugars and sweeteners have been observed to suppress the bitterness of some foods [57].

Our electrophysiological results also showed that sugar solutions inhibit the spike activity of deterrent and salt cells at low concentrations in *P. machaon*, and only at high concentrations in *P. hospiton*. Behavioural results indicate that sugar solutions dissolved in KCl are all appetitive for *P. machaon*, but only at high concentrations for *P. hospiton* (as compared with those dissolved in water). This suggests that activation of phagostimulatory cells by sugars determines a counterbalancing effect of the inhibitory input by deterrent cells, which is more relevant in *P. machaon* than in *P. hospiton* in agreement with the wider range of food acceptance by the former species. We speculate that a lateral inhibition may exists between phagostimulant and deterrent taste cells within the same sensillum, which could modulate feeding behaviour, and might be due to the presence of gap-junctions or ephaptic interactions between adjacent cells. Previous studies have reported that, when insect chemosensilla are stimulated by binary compound mixtures, the presence of one component might suppress the response to the other (suppression or hypoadditivity) or enhance it (synergism), and that the inhibitory effect could be explained by the presence of electric synapses, ephaptic interactions or antagonist interactions [23], [58], [59]. Interactions at the periphery between neurons play an important role in the food selection of herbivorous insects [3]. In fact, in oligophagous and polyphagous species, feeding is governed by the balance of phagostimolatory and deterrent inputs: input from deterrent cells may prevent the insect from feeding and the absence or decrease of a deterrent response is permissive, allowing the insect to feed [3].

*P. hospiton* and *P. machaon* are oligophagous using various plants in the Apiaceae and Rutaceae families as hosts, and larvae do not feed on any plants outside of these two plant families, although *P. machaon* feeds on two species in the Asteraceae in Alaska [60], [61]. In Sardinia, *P. hospiton* mostly uses one host plant only, *F. communis*, and lays eggs on *F. arrigonii* (narrow endemic) and *R. lamarmorae* (rare) only if *F. communis* is not available. Instead, *P. machaon* lays eggs on several Apiaceae and a few Rutaceae: this suggests that *P. hospiton* is somewhat more specialized than *P. machaon*. In herbivorous insects host specificity is determined not only by larval food acceptance but also by female oviposition preferences. The place of hatching is generally determined by female oviposition preference and, in some cases, lepidopterous larvae may have no choice but just adapt to the plant or let die. In this respect, peripheral taste sensitivity of the larvae plays an important role, as the feeding is governed by the balance between phagostimulant and phagodeterrent inputs [3].

A central pattern generator for chewing has been localized to the subesophageal ganglion. Excitatory sensory input resulting from the application of phagostimulants the mouthparts increases the frequency of the chewing rhythm which is in turn inhibited by the inputs from taste receptor cells that respond to deterrent compounds. Besides, elimination of inputs of thoracic origin causes continuous chewing movements of the mandibles [56], [62]. The source of this inhibition appears to be related to the hunger status of the larva, but also to inputs from taste receptor cells that respond to deterrent compounds. Feeding is then triggered when the net level of excitation from chemosensilla on the mouthparts surpasses a threshold of inhibition [12]. We propose that the different food choices shown by *P. hospiton* and *P. machaon* larvae may be based on qualitative and quantitative differences in the gustatory sensitivity of the GRNs in both styloconic sensilla and on the total excitatory and inhibitory input to the feeding circuitry. The excitatory inputs from phagostimulant GRNs in *P. hospiton* is higher than in *P. machaon* and this could be needed to counteract inputs from deterrent GRNs, which are quantitatively higher in *P. hospiton* than in *P. machaon*. On the other hand, *P. machaon* shows a lower sensitivity to deterrent compounds and a stronger decreasing effect by phagostimulant cells on phagodeterrent inputs with respect to *P. hospiton*; this could mean that the balance between phagostimolant and phagodeterrent inputs will more likely change in favour of an appetitive behavioural net response, thus activating the feeding circuitry and allowing the insect to potentially adapt to a wider range of host plants.

In conclusion, these findings suggest that differences in the sensitivity of the peripheral gustatory system and possible interactions between neighbouring GRNs could explain how two closely phylogenetically related species can exhibit a different food repertoire. The sensitivity differences between the two species in response to the tested compounds were not found on the basis of the spike sorting analysis: in fact, the quantitative difference in response to nicotine and both quantitative and qualitative differences in response to sugars were already identified simply by visual inspection, and confirmed by spike amplitude analysis. Instead, the hypothesis that the different feeding behaviour of the two species to disks moistened with sugar+KCl solutions may be explained by an interaction between neighbouring neurons firing spikes of similar amplitude, relies on spike sorting.

In addition, our results set the basis for investigating the response profiles to complex stimuli such as plant saps, presently being scrutinized in our lab, where the integrated response to phagostimulatory and deterrent compounds can ultimately determine whether a caterpillar eats or not a given food. Moreover, as it is known that herbivorous insects are tuned in on the chemistry of their host plants, more information about the composition of host plant foliage is also needed to better explain the functional significance of these findings in relation to the feeding behaviour of *P. hospiton* and *P. machaon* larvae.

## Supporting Information

File S1 **For each figure presented (S1–S9) the following data are provided as they are displayed by the Clampfit 10.0 software:** a) a sample of the spike discharge; b) an X-axis (time) expansion of the detail within cursors in a) positioned as indicated by Clampfit 10.0; c) spike width class frequency distribution histogram; d) table of spike width values plotted in the histogram in c). The spikes shown in b) are in red in the table. Figure S1. *P. hospiton*, lateral sensillum, Glucose 100 mM. Figure S2. *P. hospiton*, lateral sensillum, Sucrose 100 mM. Figure S3. *P. hospiton*, lateral sensillum, Inositol 10 mM. Figure S4. *P. hospiton*, medial sensillum, Inositol 10 mM. Figure S5. *P. machaon*, lateral sensillum, KCl 50 mM. Figure S6. *P. machaon*, lateral sensillum, Inositol 10 mM. Figure S7. *P. machaon*, medial sensillum, KCl 50 mM. Figure S8. *P. machaon*, medial sensillum, Glucose 100 mM. Figure S9. *P. machaon*, medial sensillum, Inositol 10 mM.
(DOC)
