# Supplementary material for: Gustatory Sensitivity and Food Acceptance in Two Phylogenetically Closely Related Papilionid Species: Papilio hospiton and Papilio machaon
Source: PLoS One. 2014 Jun 23;9(6):e100675. doi: 10.1371/journal.pone.0100675 (PMC4067346; doi:10.1371/journal.pone.0100675)
Supplement: File S1 — For each figure presented (S1–S9) the following data are provided as they are displayed by the Clampfit 10.0 software: a) a sample of the spike discharge; b) an X-axis (time) expansion of the detail within cursors in a) positioned as indicated by Clampfit 10.0; c) spike width class frequency distribution histogram; d) table of spike width values plotted in the histogram in c). The spikes shown in b) are in red in the table. Figure S1. P. hospiton, lateral sensillum, Glucose 100 mM. Figure S2. P. hospiton, lateral sensillum, Sucrose 100 mM. Figure S3. P. hospiton, lateral sensillum, Inositol 10 mM. Figure S4. P. hospiton, medial sensillum, Inositol 10 mM. Figure S5. P. machaon, lateral sensillum, KCl 50 mM. Figure S6. P. machaon, lateral sensillum, Inositol 10 mM. Figure S7. P. machaon, medial sensillum, KCl 50 mM. Figure S8. P. machaon, medial sensillum, Glucose 100 mM. Figure S9. P. machaon, medial sensillum, Inositol 10 mM. (DOC) [file pone.0100675.s001.doc]

Figure S1 (a, b)

Figure S1 (c, d)

Figure S2 (a, b)

Figure S2 (c, d)

Figure S3 (a, b)

Figure S3 (c, d)

Figure S4 (a, b)

Figure S4 (c, d)

Figure S5 (a, b)

Figure S5 (c, d)

Figure S6 (a, b)

Figure S6 (c, d)

Figure S7 (a, b)

Figure S7 (c, d)

Figure S8 (a, b)

Figure S8 (c, d)

Figure S9 (a, b)

Figure S9 (c, d)
